# SRY-box transcription factor 21 antisense divergent transcript 1: Regulatory roles and clinical significance in neoplastic conditions and Alzheimer's Disease

**DOI:** 10.7150/jca.89619

**Published:** 2023-10-02

**Authors:** Ling Lei, Guangxi Peng, Hongliang Luo, Wugen Li

**Affiliations:** 1 Department of Gastrointestinal Surgery, The Second Affiliated Hospital of Nanchang University, Nanchang 330008, Jiangxi, China.; 2 Department Prevention and Treatment Center, Jiujiang Hospital of Traditional Chinese Medicine, Jiujiang 332005, Jiangxi, China.; 3 Wart 1 of General Surgery, Yingtan People's Hospital, Yingtan 335000, Jiangxi, China.; 4 Department of Radiology, The Second Affiliated Hospital of Nanchang University, Nanchang 330008, Jiangxi, China.

**Keywords:** LncRNA, SOX21-AS1, Neoplasms, Alzheimer, Disease biomarker, Biological Functions, Clinical application

## Abstract

SRY-box transcription factor 21 antisense divergent transcript 1 (SOX21-AS1) is a multifaceted long non-coding RNA (lncRNA) that plays diverse roles in both neoplastic conditions and Alzheimer's disease. Its aberrant expression intricately regulates a wide spectrum of cellular processes, spanning from epithelial-mesenchymal transition (EMT), apoptosis, migration, metastasis, and stemness to drug resistance. SOX21-AS1 achieves these effects through its involvement in the competitive endogenous RNA (ceRNA) network, modulation of downstream genes, and regulation of critical pathways, including PI3K/AKT, Hippo, Wnt/β-catenin, and ERK signaling. Of significant clinical relevance, SOX21-AS1 expression has shown robust correlations with various clinical-pathological features. Moreover, it has demonstrated promising prognostic and diagnostic potential across a spectrum of tumors, as evidenced by existing literature and TCGA pan-cancer analyses. In Alzheimer's disease, SOX21-AS1 assumes a distinctive role. It influences neuronal viability, apoptosis, and oxidative stress by interacting with miR-107 and miR-132, and affecting the PI3K/AKT and Wnt signaling pathways. This comprehensive review sheds light on the functions of SOX21-AS1 and the regulated mechanisms underpinning its impact on neoplastic conditions and Alzheimer's disease. It underscores the clinical significance of SOX21-AS1 and positions it as a promising therapeutic target in both the oncological and neurodegenerative domains.

## Introduction

Long non-coding RNA (lncRNA) is an RNA molecule with a length exceeding 200 nucleotides, lacking protein-coding capacity 1, 2. Once considered "junk RNA," lncRNAs have been increasingly discovered with the advancement of gene sequencing technologies, and some of them have emerged as newcomers in various human diseases 3-5, playing pivotal roles in conditions including neurological disorders 6, 7 and cancer development 8, 9.

The Homo sapiens SRY-box transcription factor 21 antisense divergent transcript 1 (SOX21-AS1) is an example of an RNA gene categorized as a lncRNA. This specific gene is positioned on the 13th chromosome of the human genome, precisely at q32.1. It is composed of two exons and spans a length of 3,230 nucleotide (nt) (source: https://www.ncbi.nlm.nih.gov/gene/100507533). The resulting lncRNA from the SOX21-AS1 gene exhibits a diversity of three splice variants, ranging in size from 1565 base pairs (bp) for SOX21-AS1-202 to 3287 bp for SOX21-AS1-201 (source: https://www.ensembl.org/Homo_sapiens/Gene/Summary?g=ENSG00000227640;r=13:94703454-94803430).

SOX21-AS1, identified as a long non-coding RNA, holds notable significance in the genesis of diseases, most notably in the context of Alzheimer's disease (AD) 10-12. Its involvement is closely associated with the pathological transformations in the nervous system that arise during AD progression. Additionally, its role in driving cancer progression has recently attracted substantial attention. Across a spectrum of human tumors, including oral 13, lung 14-16, hepatocellular 17, pancreatic 18, endometrial 19, cervical 20-24, and breast cancers 25-27, as well as nephroblastoma 28, glioma 29, and osteosarcoma 30, 31, SOX21-AS1 experiences aberrant regulatory patterns. Abnormal expression of SOX21-AS1 in tumor patients has been correlated with clinicopathological characteristics and survival outcomes, encompassing aspects like lymph node metastasis, tumor dimensions, clinical stage, and overall survival. Furthermore, SOX21-AS1 assumes a pivotal function in critical biological mechanisms, including epithelial-mesenchymal transition (EMT), cell proliferation, and metastasis. Considering its significant contribution to the occurrence and progression of various diseases, SOX21-AS1 could serve as a valuable biomarker with potential applications in diagnosing, prognosticating, and devising effective therapeutic strategies for multiple pathological diseases.

Within this review, we present an encompassing synthesis of recent advancements in the understanding of the roles enacted by lncRNA SOX21-AS1 in both tumor development and Alzheimer's disease. Our focus is centered on uncovering the underlying mechanisms that drive the effects of lncRNA SOX21-AS1 in disease progression, while concurrently exploring its potential for clinical utilization across diverse malignancies as well as analyzing the prognostic and diagnostic value using The Cancer Genome Atlas (TCGA) datasets in more tumors. This review underscores the promising potential of lncRNA SOX21-AS1 as a target for therapeutic interventions applicable to a wide array of cancer forms and Alzheimer's disease.

## LncRNA SOX21-AS1 effects on neoplastic diseases

Research has explored the functions of lncRNA SOX21-AS1 across ten distinct tumor types. These have been carried out through *in vitro* and/or *in vivo* experiments, and a detailed summary of them is provided in **Table 1**.

**Figure 1:**
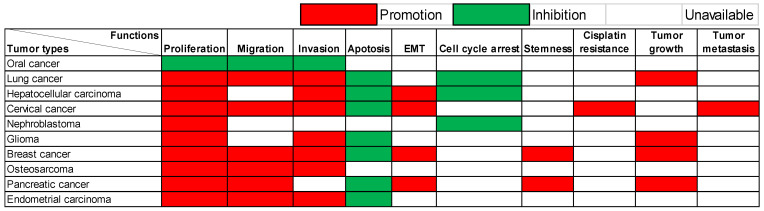
Multiple functions of lncRNA SOX21-AS1 in different human tumors verified by *in vitro* and/or *in vivo* experiments.

The multifaceted functions of this lncRNA exhibit remarkable variability contingent upon the unique context of each cancer type, as visualized in **Figure 1**. Predominantly observed in the majority of documented tumor instances, lncRNA SOX21-AS1 demonstrates an up-regulation within cancerous tissues and cell lines, effectively manifesting as a potent tumor oncogene. In detail, lncRNA SOX21-AS1 has been observed to exert influence over various critical cellular processes across these tumors. It functions as a catalyst for cell proliferation enhancement within nine different tumor types. In tandem with this, it assumes a role in inhibiting cell apoptosis across seven tumor categories. The phenomenon of EMT, pivotal in augmenting cellular capacities for invasion and metastasis, is notably influenced by lncRNA SOX21-AS1. Particularly in hepatocellular, cervical, breast, and pancreatic cancers, its overexpression triggers the promotion of EMT processes. Moreover, tumor cell migration and invasion were significantly inhibited after the knockdown of lncRNA SOX21-AS1 in six and seven tumor types, respectively, as shown in **Figure 1**. In addition, SOX21-AS1 overexpression not only leads to cell cycle arrest in the lung, hepatocellular cancers, and nephroblastoma but also contributes to the stemness in breast and pancreatic cancers. Impressively, studies employing mouse models underscore its capacity to promote tumor growth and facilitate metastasis in several kinds of tumors. However, oral cancer tissues revealed a different pattern in which the lncRNA SOX21-AS1 showed downregulation 13. LncRNA SOX21-AS1 assumes the role of a tumor suppressor, effectively inhibiting cell proliferation, migration, and invasion 13.

## ceRNA network involving lncRNA SOX21-AS1 in tumor progression

In recent times, there has been a growing exploration of competing endogenous RNAs (ceRNAs) networks, which serve as protective barriers for mRNA molecules, shielding them from miRNA-mediated inhibition 32. ceRNA regulatory networks, governed by lncRNAs, plays a pivotal role in tumor progression and development 33-37.

**Figure 2:**
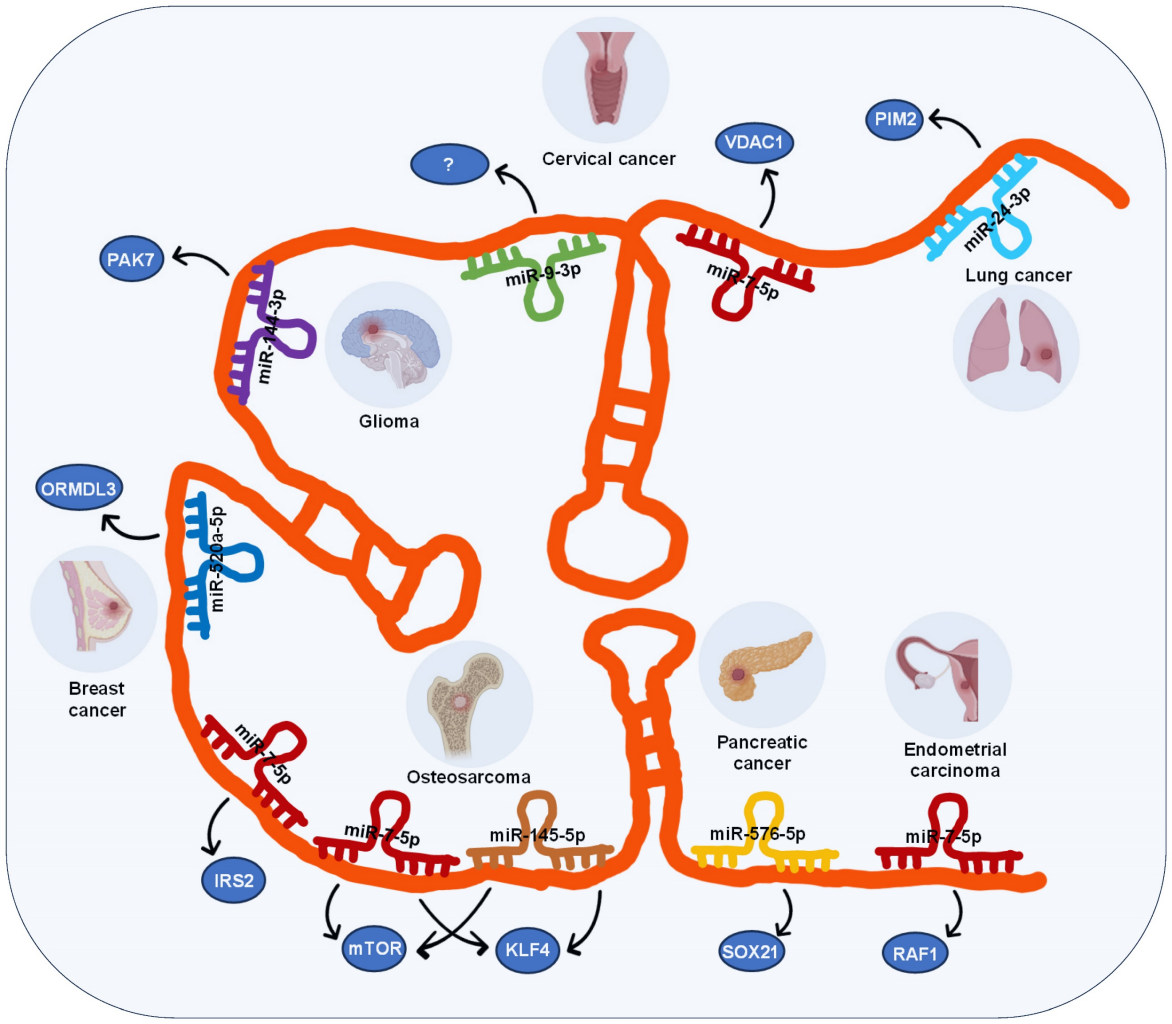
The ceRNA networks governed by lncRNA SOX21-AS1 across diverse human tumors.

In the case of lncRNA SOX21-AS1, its ceRNA network involves 7 miRNAs across 7 different types of cancers, including lung, cervical, breast, pancreatic, and endometrial carcinomas as well as glioma and osteosarcoma (**Figure 2**), these miRNAs include miR-24-3p, miR-7-5p, miR-9-3p, miR-144-3p, miR-520a-5p, miR-145-5p, and miR-576-5p.

LncRNA SOX21-AS1 modulates its regulatory function in various tumors through competitive binding with diverse miRNAs. For instance, in lung cancer 14, lncRNA SOX21-AS1 functions as a sponge for miR-24-3p, leading to the upregulation of PIM2, which facilitates their proliferation, migration, and invasion. In glioma 29, lncRNA SOX21-AS1 acts as a sponge for miR-144-3p, resulting in the upregulation of PAK7, which induces tumor cell proliferation, migration, and invasion, while inhibiting apoptosis. In pancreatic cancer 18, lncRNA SOX21-AS1 acts as a sponge for miR-576-5p, resulting in the upregulation of SOX21, and promoting cancer progression.

Interestingly, lncRNA SOX21-AS1 also participates in the development of the same tumor by engaging with multiple miRNAs. For example, in cervical tumors, two different ceRNA mechanisms involving lncRNA SOX21-AS1 have been identified: it can promote cell proliferation, migration, and invasion of cervical tumor cells by targeting miR-7-5p/VDAC1 axis and miR-9-3p 23. Similarly, in osteosarcoma 30, 31, lncRNA SOX21-AS1 interacts with two miRNAs (miR-7-5p and miR-145-5p) subsequently upregulating the expression of three target genes (IRS2, mTOR, KLF4), thereby accelerating the progression and metastasis of osteosarcoma.

Moreover, lncRNA SOX21-AS1 is involved in the advancement of diverse tumors through its interaction with the same miRNA. Specifically, lncRNA SOX21-AS1 competes for binding with miR-7-5p, resulting in the increased expression of VDAC1 in cervical cancer 23, IRS2 and mTOR in osteosarcoma 30, 31, RAF1 in endometrial cancer 19, thereby promoting tumor progression.

## Downstream genes regulated by lncRNA SOX21-AS1 in tumors

**Figure 3:**
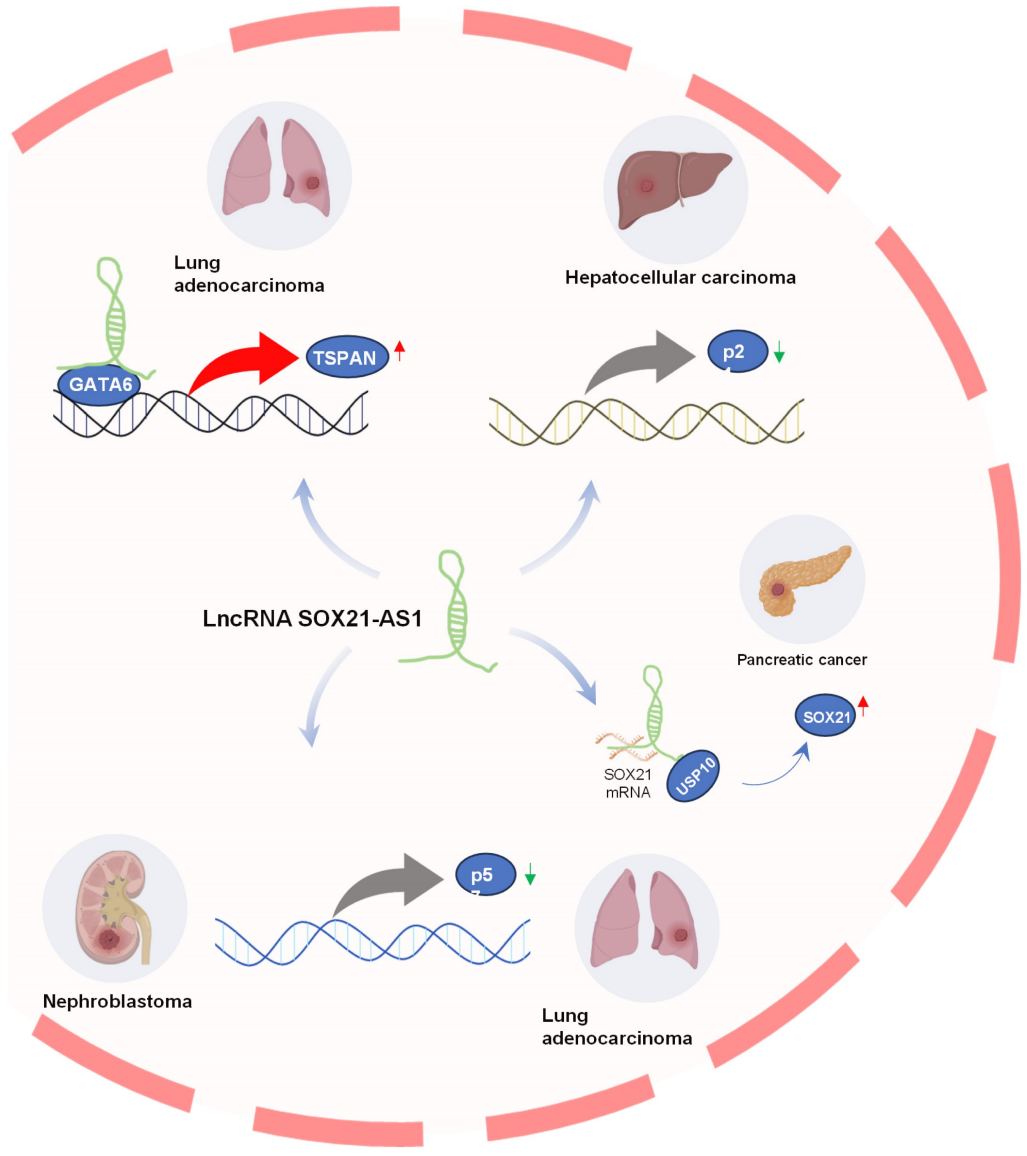
Downstream genes regulated by lncRNA SOX21-AS1. SOX21-AS1 interacts with various genes to promote tumor development, including upregulating TSPAN8 in lung adenocarcinoma, silencing p21 in hepatoma progression, regulating p57 in nephroblastoma and LUAD, and post-transcriptionally influencing SOX21 expression in pancreatic cancer.

LncRNAs exhibit regulatory effects on neighboring genes (cis) by mechanisms such as transcriptional interference and can also influence distant genes (trans) by modulating epigenetic modifications 38-40. Downstream genes regulated by lncRNA SOX21-AS1 in tumors are shown in **Figure 3**. In pancreatic cancer, Yu et al. 18 found that SOX21-AS1 positively regulated its nearby gene SOX21 at post-transcriptional level to promote tumor progression. LncRNA SOX21-AS1 acts as a molecular sponge, efficiently sponging SOX21 mRNA to enhance its stability and expression, and also recruits USP10, leading to deubiquitination and subsequent stabilization of SOX21 protein. Moreover, STAT6 could transcriptionally activate SOX21-AS1 and SOX21 expression.

TSPAN8 plays a role in the regulation of tumor cell growth and motility 41, 42. It has also been implicated in promoting metastasis 43, 44 and angiogenesis 45. In lung adenocarcinoma 15, SOX21-AS1 was found to interact with GATA6, which is bound to the promoter region of TSPAN8, consequently promoting TSPAN8 expression. This heightened expression of TSPAN8 further amplified tumorigenesis in lung adenocarcinoma.

The p21 gene, located downstream of the p53 gene, serves as a tumor suppressor gene, encoding a vital cell cycle regulatory protein 46. Disrupted expression of p21 can perturb the regulation of cell proliferation and differentiation, ultimately contributing to the development of malignant tumors 47, 48. Notably, in hepatocellular carcinoma (HCC) 17, SOX21-AS1 was implicated in the epigenetic silencing of p21 by recruiting EZH2 to the p21 promoter region, thereby promoting the progression of hepatoma.

p57, also known as CDKN1C, functions as a potent inhibitor of multiple G1 cyclin/Cdk complexes and serves as a negative regulator of cell proliferation 49, 50, it also regulates apoptosis, differentiation, development, and migration. It was considered a tumor suppressor candidate in various tumorigenesis processes 51-53. In lung cancer, SOX21-AS1 was found to promote cancer cell proliferation by repressing p57 transcription through its interaction with associated proteins 16. Additionally, the knockdown of SOX21-AS1 led to the suppression of nephroblastoma cell proliferation and colony formation, triggering cell-cycle arrest via the upregulation of p57 expression 28.

## Tumor-related signaling pathways influenced by lncRNA SOX21-AS1

**Figure 4:**
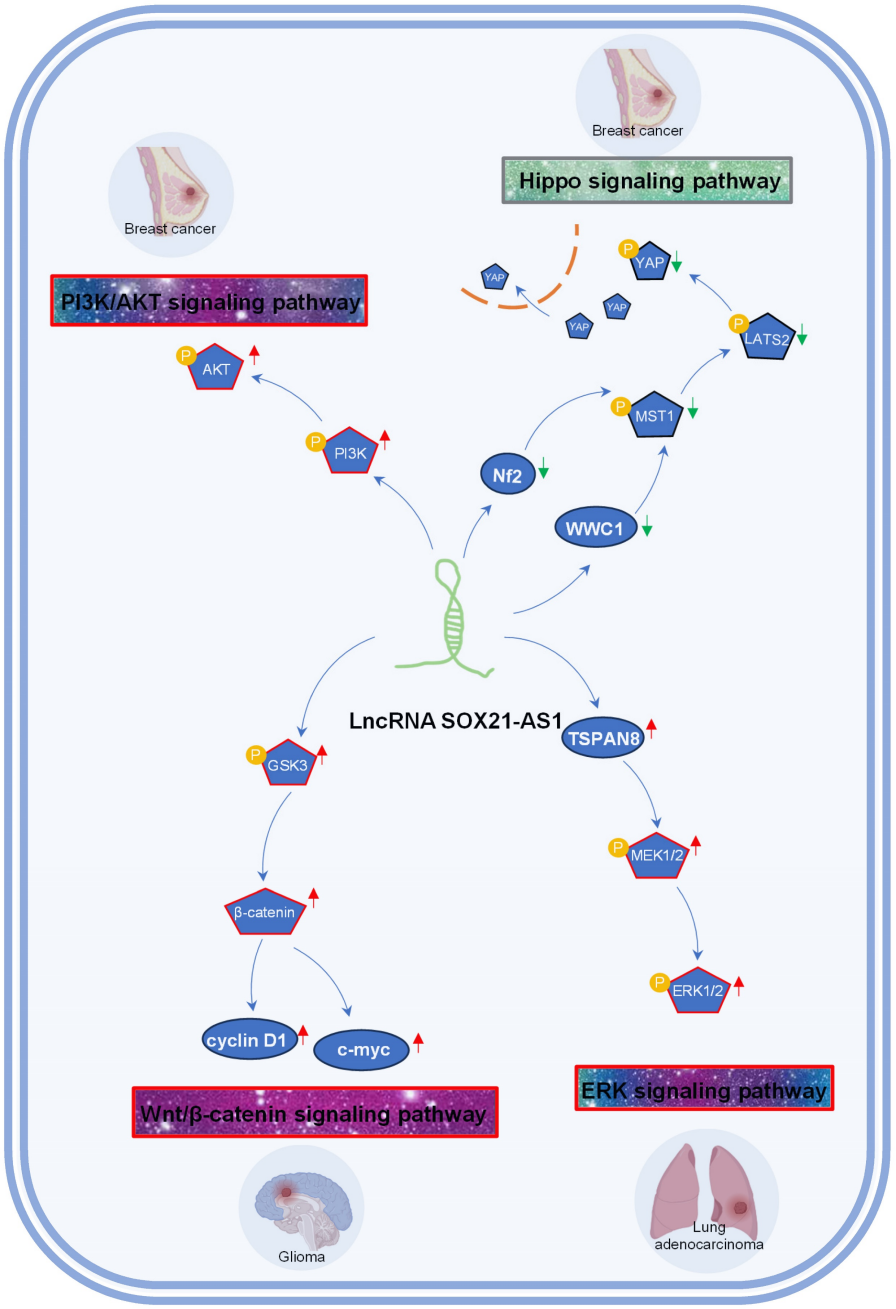
Tumor-associated signaling pathways influenced by lncRNA SOX21-AS1 in breast cancer, lung adenocarcinoma, and glioma. SOX21-AS1 is implicated in promoting tumorigenesis by activation of PI3K/AKT signaling pathway, Wnt/β-catenin signaling pathway, ERK signaling pathway, and inactivation of Hippo signaling pathway.

A growing body of evidence suggests that lncRNAs contribute to the regulation of diverse signaling pathways 54-57, offering new avenues for targeted therapies. Presently, SOX21-AS1 has emerged as a key player in driving tumorigenesis by activating several signaling pathways, such as Wnt/β-catenin, ERK, and PI3K/AKT, while inactivating the Hippo signaling pathway. SOX21-AS1's engagement with these signaling cascades implies a far-reaching influence on tumor cell behavior and potential therapeutic strategies, as depicted in **Figure 4.**

The phosphoinositide 3-kinase (PI3K) and AKT pathway is a critical regulator of cell growth, motility and metabolism 58, 59. Activation of PI3K/AKT signaling contributes to tumor recurrence, metastasis and drug resistance 60-62. In breast cancer 26, SOX21-AS1 overexpression resulted in the increased phosphorylation of PI3K and AKT, consequently fostering the proliferation, invasion, migration, and EMT of breast cells by activating the PI3K/AKT pathway.

The Hippo signaling pathway plays a crucial role in organ size control and tissue regeneration. The Hippo signaling pathway inhibits cell proliferation and growth and promotes apoptosis, and inactivation of the Hippo pathway is often observed during cancer development. In breast cancer 25, SOX21-AS1 has been linked to the inactivation of the Hippo pathway, an event characterized by the disruption of the Hippo core kinase cascade. This disruption includes the increased nuclear localization of YAP and a concurrent reduction in pYAP levels. Elevated SOX21-AS1 expression leads to a notable decrease in the expression levels of critical Hippo pathway components, namely WWC1, Nf2, pMST1, pLATS2, and pYAP 25. Consequently, SOX21-AS1 overexpression fosters stemness within the cancer cells, intensifying their proliferation, migration, and invasion capabilities through the inhibition of the Hippo signaling pathway 25.

The Wnt/β-catenin pathway regulates cell fate, tissue homeostasis, and embryonic development 63, 64. Activation of this pathway, including the accumulation of β-catenin, leads to its translocation into the nucleus, where it forms complexes with transcription factors to activate target genes 65. In glioma 29, SOX21-AS1 has been shown to promote the activation of the Wnt/β-catenin signaling, propelling cancer cells toward a more aggressive phenotype characterized by enhanced proliferation and invasiveness.

The ERK signaling pathway is a master regulator of cellular behavior and fate, encompassing processes such as cell proliferation, survival, and migration 66, 67. In the context of lung adenocarcinoma 15, the inhibition of SOX21-AS1 led to a reduction in MEK1/2 and ERK1/2 phosphorylation. This could be rescued by the overexpression of TSPAN8. Notably, SOX21-AS1 was confirmed to enhance lung cancer cell proliferation, migration, and invasion by activating the ERK signaling pathway through its interaction with TSPAN8.

## LncRNA SOX21-AS1 effects on Alzheimer's disease

**Figure 5:**
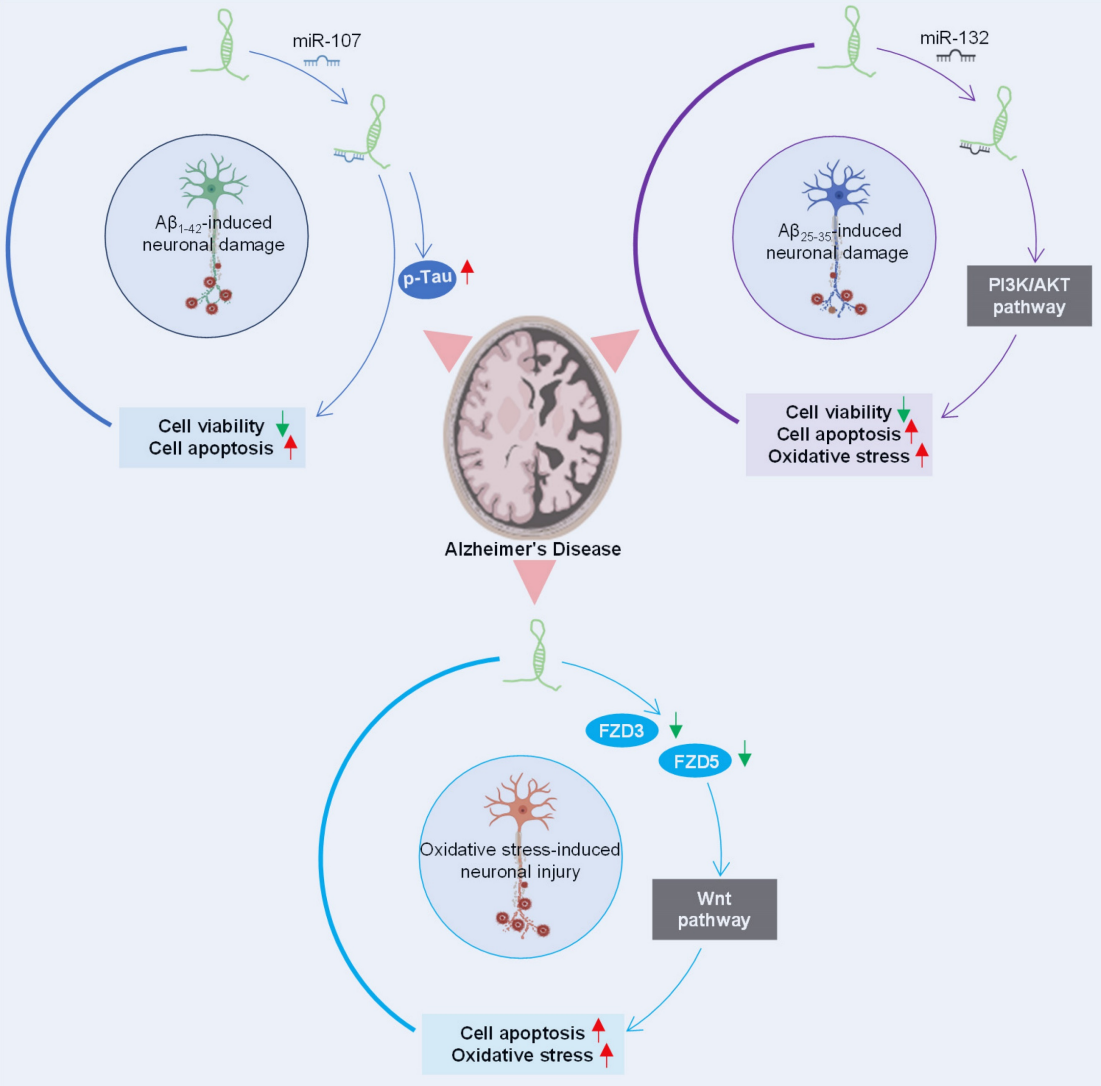
Regulatory network orchestrated by SOX21-AS1 in Alzheimer's disease pathogenesis. SOX21-AS1 decreases the levels of miR-107 and miR-132 after sponging with them and influences the inactivation of the PI3K/AKT pathway and Wnt signaling pathway, affecting neuronal viability, apoptosis, and oxidative stress. The potential therapeutic significance of silencing SOX21-AS1 is highlighted as a strategy for mitigating neurodegenerative damage in Alzheimer's disease.

The role of lncRNA SOX21-AS1 in the pathogenesis of Alzheimer's disease has gained much attention. Multiple studies have delved into its potential impact on the pathophysiology of this neurodegenerative disorder (**Figure 5**). The silencing of lncRNA SOX21-AS1 emerges as a promising avenue for therapeutic interventions aimed at mitigating neurodegenerative damage in Alzheimer's disease.

In a study conducted by Xu et al. 11, SOX21-AS1 was identified as a miR-107 sponge, revealing an inverse correlation between the expression levels of miR-107 and SOX21-AS1. The knockdown of SOX21-AS1 effectively reversed the viability and apoptosis alterations induced by miR-107 inhibition in Aβ_1-42_-treated SH-SY5Y and SK-N-SH cells.

Additionally, research by Gu et al. 10 highlighted SOX21-AS1's role in exacerbating Aβ_25-35_-induced neuronal cell injury by targeting miR-132 and impeding the PI3K/AKT pathway. It's noteworthy that the PI3K/AKT signaling pathway, which exhibits protective properties in neurodegenerative diseases 68, becomes inactivated in Alzheimer's disease but is conversely activated in human cancers 69.

Furthermore, investigations by Zhang et al. 12 suggested that the suppression of SOX21-AS1 could ameliorate neuronal oxidative stress injury in an Alzheimer's disease mouse model. SOX21-AS1 overexpression was found to drive the progression of Alzheimer's disease, elevating neuronal oxidative stress levels and promoting neuronal apoptosis. These effects are mediated through downregulation of FZD3/5 and subsequent inactivation of Wnt signaling, whose activation is neuroprotective in neurodegenerative diseases 70.

## Potential application of lncRNA SOX21-AS1 in human disease

SOX21-AS1 has demonstrated potential applications in disease diagnosis, prognosis and therapy. The clinical value of SOX21-AS1 as an important tumor marker has been reported by many studies (**Table 2**). SOX21-AS1 may be a promising therapeutic target in clinical settings, including several oncological diseases and Alzheimer's disease. Understanding how SOX21-AS1 can be harnessed in clinical practice may open the door to innovative diagnostic tools, therapeutic interventions, and personalized treatment strategies, ultimately improving patient care and treatment outcomes.

## LncRNA SOX21-AS1 as a diagnostic marker

The clinical utility of lncRNA SOX21-AS1 as a diagnostic marker has been reported in breast cancer 26, exhibiting an impressive area under the curve (AUC) of 0.930 in the receiver operating characteristic (ROC) curve. Similarly, its diagnostic potential has been demonstrated in endometrial carcinoma, where the AUC of the ROC curve was shown to be 0.639 19.

**Figure 6:**
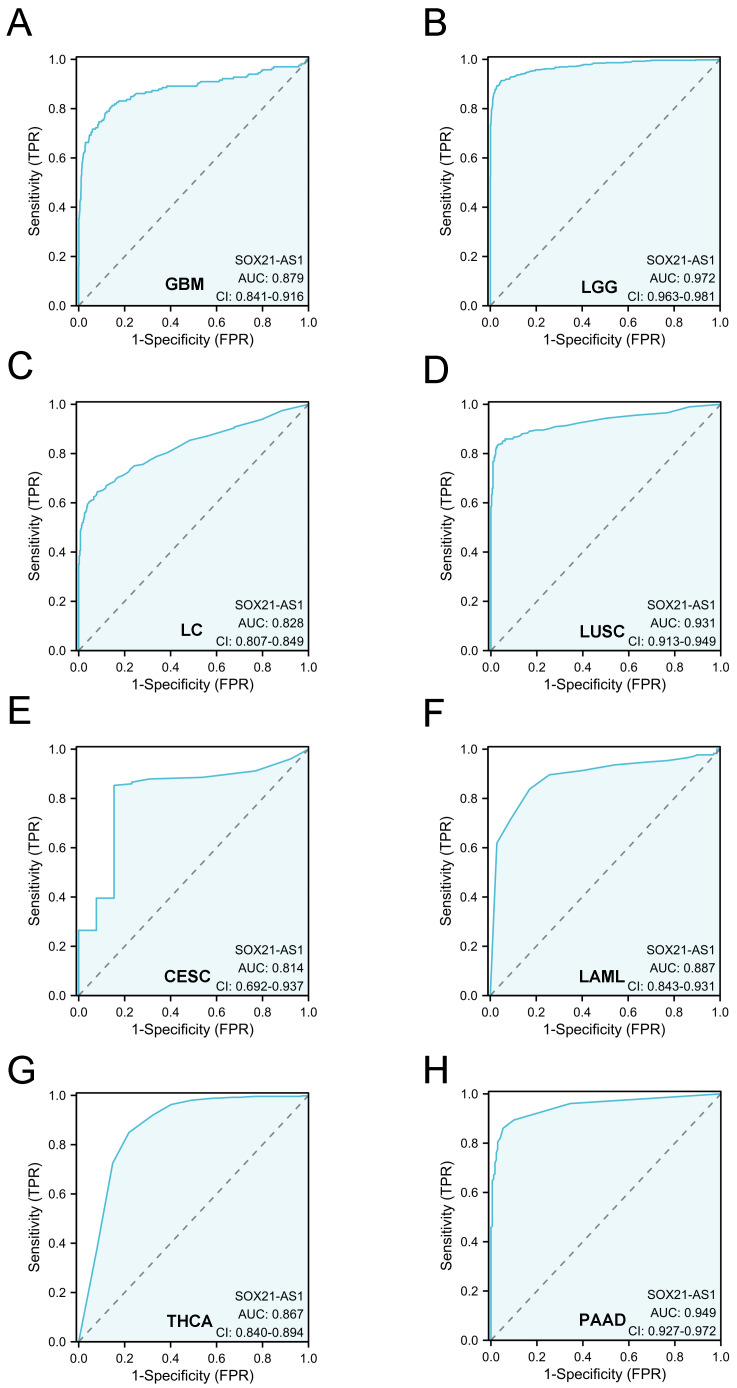
The strong diagnostic potential of lncRNA SOX21-AS1 in distinguishing tumor from normal tissue across multiple tumor types. SOX21-AS1 expression demonstrates substantial diagnostic utility, with an impressive area under the ROC curve exceeding 0.8, observed in GBM (A), LGG (B), LC (C), LUSC (D), CESC (E), LAML (F), THCA (G), and PAAD (H).

To expand this assessment, we conducted ROC curve analyses across a diverse spectrum of cancers using TCGA data (**Figure 6**). Remarkably, these analyses illuminated SOX21-AS1's potential as a robust diagnostic biomarker across numerous cancer types. Particularly noteworthy is its significance in glioblastoma multiforme (GBM), low-grade glioma (LGG), lung cancer (LC), lung squamous cell carcinoma (LUSC), cervical squamous cell carcinoma and endocervical adenocarcinoma (CESC), acute myeloid leukemia (LAML), thyroid carcinoma (THCA), and pancreatic adenocarcinoma (PAAD), where the AUC exceeded 0.8. All the above results indicated that SOX21-AS1 holds promise as a diagnostic predictor in diverse malignancies.

## LncRNA SOX21-AS1 as a prognostic marker

SOX21-AS1 expression levels have been found to correlate with clinical features and patient prognosis in various tumors (**Table 2**). For instance, in oral cancer 13, SOX21-AS1 is a protective lncRNA as it was down-regulated in oral cancer tissues, and its low expression was found to be advanced AJCC pathological stage, and higher T classification and low expression of SOX21-AS1 indicated poor prognosis regarding disease-specific survival (DSS), disease-free survival (DFS), while in most of the reported tumors, SOX21-AS1 is a risk lncRNA as it was significantly up-regulated in cancer tissues, including lung cancer, HCC, glioma, breast cancer, nephroblastoma, osteosarcoma, pancreatic cancer, and endometrial carcinoma, and its high expression was found to be unfavorable clinical outcomes, such as larger tumor size, advanced TNM stage, and shorter survival times.

**Figure 7:**
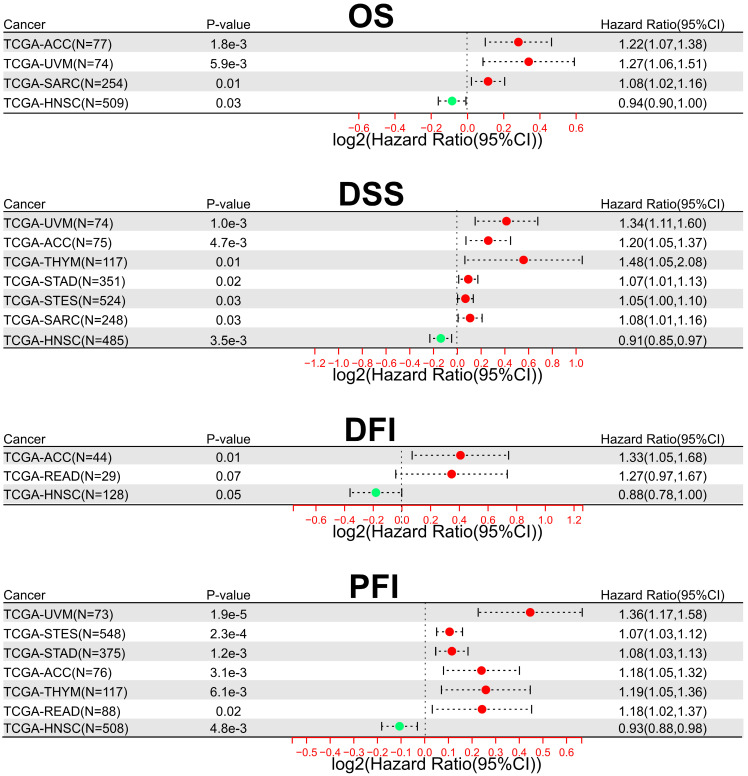
The prognostic significance of SOX21-AS1 extends beyond the tumors previously documented in publications. Overexpression of SOX21-AS1 indicates shorter OS (A), worse DSS (B), inferior DFI (C), and adverse PFI (D) across multiple malignancies after TCGA analysis.

In addition, to comprehensively evaluate the prognostic significance of SOX21-AS1 in pan-cancer, we conducted an extensive analysis using publicly available datasets from TCGA. Regarding overall survival (OS) (**Figure 7A**), we observed that SOX21-AS1 overexpression was associated with an unfavorable prognosis in adrenocortical carcinoma (ACC), uveal melanoma (UVM), and sarcoma (SARC). Conversely, it acted as a favorable predictor in head and neck squamous cell carcinoma (HNSC). In terms of DSS (**Figure 7B**), high expression of SOX21-AS1 indicated a poor prognosis in ACC, UVM, stomach adenocarcinoma (STAD), thymoma (THYM), stomach and esophageal carcinoma (STES), and SARC, whereas a good prognosis in HNSC. Furthermore, in disease-free interval (DFI) analysis (**Figure 7C**), SOX21-AS1 overexpression was indicative of a detrimental prognosis in ACC and rectum adenocarcinoma (READ). Conversely, it served as a propitious predictive factor in HNSC. In terms of progression-free interval (PFI) (**Figure 7D**), elevated expression of SOX21-AS1 was associated linked to a dismal outcome in ACC, UVM, THYM, STAD, STES, and READ. In contrast, it functioned as a promising favorable predictor in HNSC. These findings underscore the potential of SOX21-AS1 as a prognostic biomarker in various tumor types, highlighting its value in assessing patient outcomes and tailoring personalized treatment strategies.

Notably, SOX21-AS1-related prognostic models may also be useful tools for assessing the prognosis and providing new ideas for the treatment of tumors. For instance, in cervical cancer 20, a risk signature comprising eight cuproptosis-related lncRNAs (AL441992.1, SOX21-AS1, AC011468.3, AC012306.2, FZD4-DT, AP001922.5, RUSC1-AS1, AP001453.2) has been developed, enabling the prediction of prognosis and immunotherapeutic responses. Furthermore, a prognostic signature model linked to m6A-related lncRNAs (AC005229.3, SOX21-AS1, AL133523.1, and AC004847.1) has been constructed for glioblastoma multiforme 71, offering insights into prognosis and immunomodulatory effects. Additionally, a survival and prognosis model, utilizing a set of 12 m6A/m5C/m1A/m7G-related genes (AL080276.2, AC092111.1, SOX21-AS1, DNAJC9-AS1, AC025171.1, AL356019.2, AC017104.1, AC099850.3, UNC5B-AS1, AC006064.2, AC010319.4, and AC016822.1), has demonstrated robust independent prediction capabilities for glioma patients 72. Furthermore, in glioblastoma 73, a lncRNA risk score derived from five lncRNAs (RP6-99M1.2, SOX21-AS1, CTD-2127H9.1, RP11-375B1.3, and RP3-449M8.9) has been established to predict survival independently, transcending the influence of other markers. These advancements emphasize the potential of SOX21-AS1-associated prognostic models as valuable tools for prognosis assessment and the development of customized cancer treatments.

## LncRNA SOX21-AS1 is a promising disease therapy target

**Figure 8:**
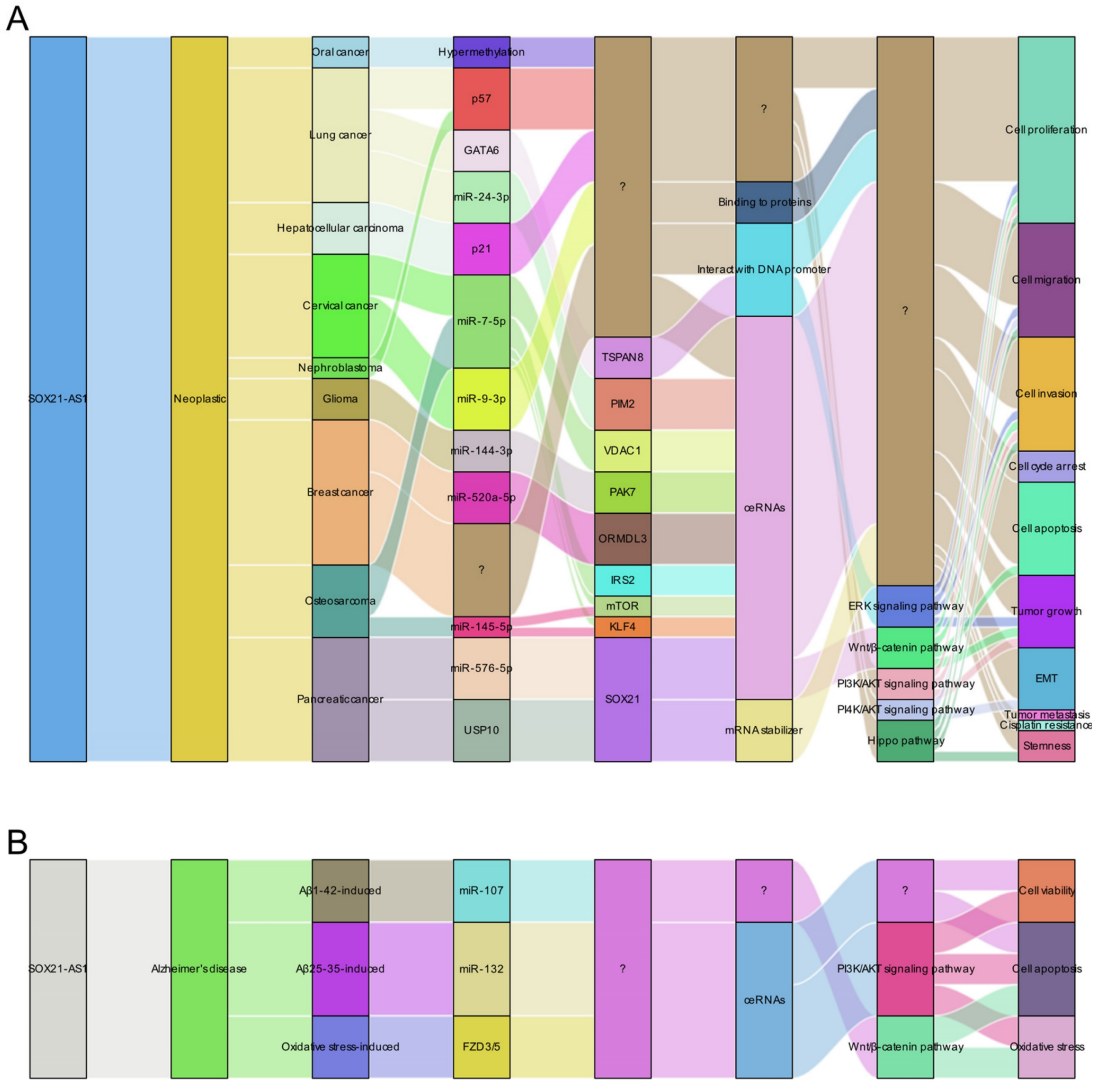
Molecular insights into the role of lncRNA SOX21-AS1 as a master regulator of cellular processes in pathological conditions. This figure presents a summary of the multifaceted molecular mechanisms by which lncRNA SOX21-AS1 exerts its pivotal role in regulating cellular processes in neoplastic conditions (A) and Alzheimer's disease (B). lncRNA SOX21-AS1 exerts its influence on cellular activities to contribute to the progression of human disease through several intricate mechanisms, including functioning as a ceRNA within networks, interacting with proteins and promoters, and serving as an mRNA stabilizer. Additionally, it plays an important role in the regulation of the signaling pathways.

**Figure 9:**
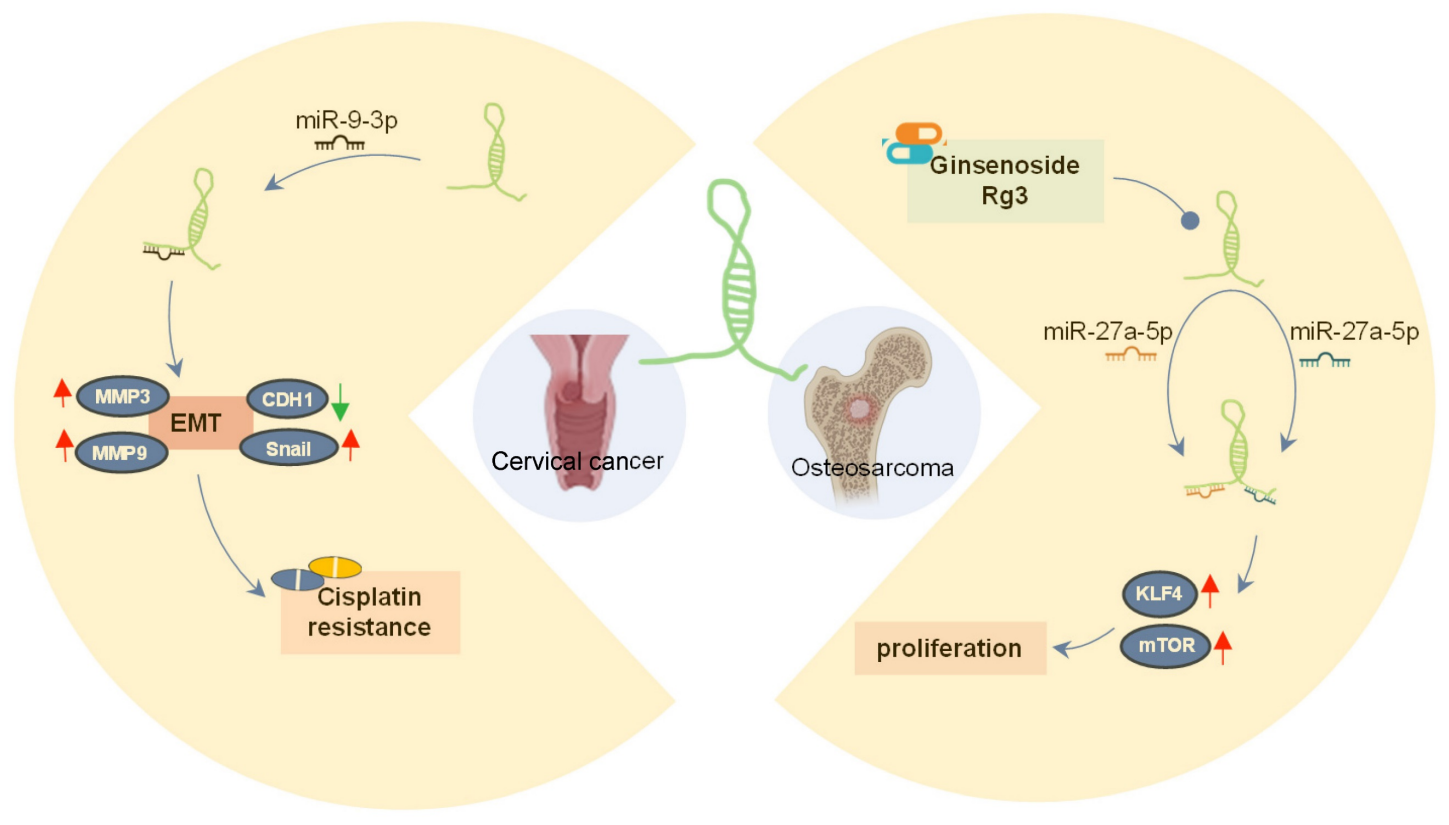
The role of lncRNA SOX21-AS1 in cancer drugs. In cervical tumors, lncRNA SOX21-AS1 contributes to heightened resistance against cisplatin. In osteosarcoma, the effectiveness of cancer cells is compromised by Ginsenoside Rg3, which inhibits the expression of lncRNA SOX21-AS1, consequently impeding cancer cell proliferation.

SOX21-AS1 has emerged as a prospective therapeutic target in both neoplastic conditions and Alzheimer's disease due to its intricate regulatory mechanisms (**Figure 8A-B**). In tumors (**Figure 8A**), SOX21-AS1 interacts with DNA, proteins, and RNA molecules, playing a crucial role in fundamental cellular processes such as cell viability, apoptosis, migration, and invasion. Operating as a ceRNA, SOX21-AS1 disrupts microRNA-mediated gene suppression, leading to the upregulation of target genes and exerting a broader regulatory influence. It also modulates downstream genes, including p21 and p57. Notably, SOX21-AS1 is involved in critical signaling pathways like PI3K/AKT, ERK, and Wnt/β-catenin. Impressively, in cervical tumors, SOX21-AS1 contributes to enhanced resistance against cisplatin (**Figure 9**). In osteosarcoma, ginsenoside Rg3 can suppress cancer cell proliferation by inhibiting SOX21-AS1, suggesting the potential to improve the effectiveness of existing cancer treatment strategies (**Figure 9**). In Alzheimer's disease (**Figure 8B**), SOX21-AS1 reduces the levels of miR-107 and miR-132 after interacting with them, influencing the inactivation of the PI3K/AKT pathway and the Wnt signaling pathway. This, in turn, affects neuronal viability, apoptosis, and oxidative stress. Silencing SOX21-AS1 holds promise as a strategy for alleviating neurodegenerative damage. In summary, the multifaceted functions of SOX21-AS1 position it as a promising target for disease therapy, offering potential avenues for intervention in various pathological conditions.

## Future perspectives and conclusion

LncRNAs have emerged from the shadows of genetic obscurity to become key players in a variety of human diseases 74-76, including non-neoplastic and neoplastic diseases. Among these, SOX21-AS1 stands out as a multifaceted lncRNA with profound implications for both neoplasms and Alzheimer's disease. In this comprehensive review, we elucidate the complex regulatory mechanisms by which SOX21-AS1 functions in these diseases and explore its potential for clinical application.

In the context of neoplastic conditions, extensive research has uncovered the versatile role of SOX21-AS1 across various tumor types. It acts as a potent oncogene in most tumors 14, 15, 17-19, 21, 23, 25-31, 77 but might serve as a tumor suppressor in head and neck squamous cell carcinomas (HNSCCs), such as oral cancer 13. The disparity in prognosis observed in HNSCCs, as opposed to other tumor types, could potentially be attributed to the unique biology of HNSCCs, the specific molecular characteristics of SOX21-AS1 in HNSCCs, and the intricate interplay between these factors and the tumor microenvironment. Further research is imperative to unravel the underlying mechanisms driving these distinctions and to gain deeper insights into the role of SOX21-AS1 in HNSCCs in comparison to other cancer types.

Generally, SOX21-AS1 overexpression promotes cell proliferation, inhibits apoptosis, induces EMT, facilitates migration, invasion, and metastasis, and even enhances stemness in cancer cells. Impressively, SOX21-AS1 is not restricted to a single mode of action; it affects tumor progression through multiple mechanisms, including ceRNA networks, downstream genes, and tumor-associated signaling pathways. Furthermore, SOX21-AS1 exhibits the potential to serve as a diagnostic and prognostic marker in cancer. Its differential expression in cancer tissues correlates with clinicopathological features and patient survival outcomes, making it a valuable tool for patient stratification and personalized treatment strategies. Notably, prognostic models incorporating SOX21-AS1 have shown promise in assessing patient outcomes and guiding clinical decisions.

In Alzheimer's disease 10-12, SOX21-AS1 also plays an important role. It interacts with specific miRNAs (miR-107 and miR-132), affecting the inactivation of PI3K/AKT and Wnt signaling pathways. These interactions lead to changes in neuronal viability, apoptosis, and oxidative stress, ultimately leading to neurodegenerative damage. Silencing of SOX21-AS1 might be a promising therapeutic strategy to mitigate these deleterious effects.

Looking ahead, the multifaceted functions of SOX21-AS1 offer exciting prospects for future research and clinical applications. SOX21-AS1's role varies across different tumor types, and further investigations are essential to comprehend its regulatory mechanisms in different solid tumors, including head and neck cancers. Additionally, various types and mechanisms of drug resistance are prevalent in different tumors, and current research on SOX21-AS1's involvement in drug resistance is primarily limited to cervical tumors. Further exploration is necessary to elucidate how SOX21-AS1 contributes to drug resistance or potentially regulates radiation sensitivity. The expression of SOX21-AS1 in liquid tissue samples, such as serum, and its potential value in the early diagnosis of tumor patients are still completely unknown. Investigating the clinical utility of SOX21-AS1 in early tumor diagnosis and monitoring is warranted. In the context of Alzheimer's disease, the precise mechanisms through which SOX21-AS1 regulates relevant pathways remain incompletely understood and require further clarification. Furthermore, SOX21-AS1 might engage in more extensive ceRNA networks in Alzheimer's disease, which also demands more investigations.

In conclusion, lncRNA SOX21-AS1 is an emerging star with significance in cancer and Alzheimer's disease. Its intricate regulatory roles and potential clinical value make it a promising candidate for disease therapy. SOX21-AS1 may serve as an effective diagnostic tool, prognostic model, and therapeutic intervention for various pathological conditions. As research in this domain advances, SOX21-AS1 is poised to deepen our comprehension of related disease mechanisms and emerge as a novel biomarker and therapeutic target with far-reaching implications for the future.

## Figures and Tables

**Table 1:** Functions and regulatory mechanisms of lncRNA SOX21-AS1 investigated through *in vitro* and/or *in vivo* experiments across various human cancers.

Tumor type	Cell line	Expression in cancer cell lines	Animal model	Regulatory mechanism	Effects *in vitro*	Effects *in vivo*	Signaling pathway	Treatment
Oral cancer 13	Human tongue squamous cell carcinoma cell lines: SAS and CAL27	-	-	Hypermethylation	Proliferation, migration, invasion	-	-	-
Lung adenocarcinoma 16	Lung adenocarcinoma cell lines (A549, SPC-A1, NCI-H1299, NCI-H1650, PC9, H1975), lung squamous carcinomas cell lines (H1703, SK-MES-1, H226), and a normal human bronchial epithelial cell line (16HBE)	-	Five-week-old male athymic BALB/c nude mice	SOX21-AS1 /p57	Proliferation, cell cycle arrest, cell apoptosis	Tumor growth	-	-
Lung adenocarcinoma 15	Lung cancer cell lines (SK-MES-1, H226, A549, H1299, H1975, H460, H661) and human bronchial epithelial cell line (16HBE)	Up- regulated	Xenograft tumor model (BALB/c nude mice, 4-5 week-old)	SOX21-AS1/ GATA6/TSPAN8	Proliferation, migration, invasion	Tumor growth	ERK signaling pathway	-
Lung cancer 14	Lung cancer cell lines(H125, A549, NCI-H23, HCC827,NCI-H1299), and normal human lung epithelial BEAS-2B cells	Up- regulated	Xenograft tumor model	SOX21-AS1/ miR-24-3p/PIM2	Proliferation, apoptosis, migration, invasion	Tumor growth	-	-
Hepatocellular carcinoma 17	Hepatocellular carcinoma cell lines (Hep3B, LM3, MHHC97H, HepG2 and Huh7) and the normal liver epithelial cell line (LO2)	Up- regulated	-	SOX21-AS1 /p21	Proliferation, cell cycle arrest, apoptosis, cell invasion, EMT	-	-	-
Cervical cancer 23	Cervical cancer cell lines (C33A, SiHa, SW756, Caski, HeLa) and noncarcinoma cervical epithelial cell line (Ect1/E6E7)	Up- regulated	-	SOX21-AS1/ miR-7-5p/VDAC1	Proliferation, apoptosis, migration, invasion	-	-	-
Cervical cancer 21	SiHa and Siha-DDP cells	-	Female BALB/c mice (4 weeks old, six in each group)	SOX21-AS1/miR-9-3p	Invasion, migration, apoptosis, EMT	Tumor metastasis	-	cisplatin resistance
Cervical cancer 20	Ca Ski cells and Ect1/E6E7 cells	Down- regulated	-	-	-	-	-	-
Nephroblastoma 28	Nephroblastoma cell lines (WiT49 and WT-CLS1) and normal human embryonic kidney cell line (HEK293)	Up- regulated	-	SOX21-AS1 /p57	Proliferation, cell-cycle arrest	-	-	-
Glioma 29	Glioma cell lines (U251, U118, LN229, U87, SHG44) and normal human astrocytes (NHA)	Up- regulated	Six-week-old male nude mice	SOX21-AS1/ miR-144-3p/PAK7	Proliferation, invasion, apoptosis	Tumor growth	Wnt/ β-catenin pathway	-
Triple-negative breast cancer 27	TNBC cells (MDA-MB-231, BT-549, MDA-MB-436, MDA-MB-468) and normal human mammary gland cell (MCF-10A)	Up- regulated	-	SOX21-AS1/ miR-520a-5p/ORMDL3	Proliferation, migration, invasion, apoptosis, EMT	-	-	-
Breast cancer 26	Breast cancer cells (MCF-7, BT-20, MDA-MB-231, MCF-10A) and normal breast epithelial cells Hs 278Bst	Up- regulated	Female BALB/c nude mice	-	Proliferation, invasion, migration, EMT	Tumor growth	PI3K/AKT signaling pathway	-
Breast cancer 25	Breast cancer cell lines (MCF-7, MDA-MB-231) and normal human breast epithelial cell line (MCF-10A)	Up- regulated	-	-	Stemness, proliferation, migration, invasion	-	Hippo pathway	-
Osteosarcoma 31	osteosarcoma cell lines (Saos-2, U2OS, and 143B) and normal human osteoblasts (NHOst)	Up- regulated	-	SOX21-AS1/ miR-7-5p/IRS2	Proliferation, migration, invasion	-	-	-
Osteosarcoma 30	osteosarcoma cell line: 143B cells	-	-	SOX21-AS1/ miR-7-5p/ miR-145-5p/mTOR/KLF4	Proliferation	-	-	Ginse-noside Rg3
Pancreatic cancer 18	Pancreatic cancer cells (CFPAC-1, Capan-1, BxPc3, PANC-1, SW1990) and normal pancreatic duct epithelial cells HPDE6c7	Up- regulated	Xenograft tumor model nude mice (4-6 weeks old)	STAT6/ SOX21-AS1 /miR-576-5p/SOX21/ USP10	Proliferation, migration, apoptosis, EMT, stemness	Tumor growth	-	-
Endometrial carcinoma 19	Endometrial carcinoma cell lines (RL95-2, HEC-1A, Ishikawa, AN3CA) and endometrial epithelial cells	Up- regulated	-	miR-7-5p/RAF1	Proliferation, migration, invasion, apoptosis	-	-	-

EMT: Epithelial to mesenchymal transition; TNBC: Triple-negative breast cancer

**Table 2:** Associations among expression levels of lncRNA SOX21-AS1, tumor types, clinical features, and prognostic outcomes.

Tumor type	Expression in tumor tissue	Clinical feature	Prognosis	Methods for survival analysis	Indicators for poor survival	Diagnosis
Oral cancer 13	Down- regulated	AJCC pathological stage, T classification	DSS, DFS	K-M plot; Univariate/ Multivariate analysis	Low expression	-
Lung adenocarcinoma 16	Up- regulated	TNM stage, tumor size	OS	K-M plot	High expression	-
Lung adenocarcinoma 15	Up- regulated	TNM stage	-	-	-	-
Lung cancer 14	Up- regulated	-	-	-	-	-
Hepatocellular carcinoma 17	Up- regulated	Tumor size, Edmondson-Steiner grade, vascular invasion, cirrhosis	OS	K-M plot	High expression	-
Cervical cancer 24	Up- regulated	-	OS	K-M plot	Low expression	-
Cervical cancer 23	Up- regulated	FIGO stage, lymph node metastasis, invasion depth	OS	K-M plot; Univariate/ Multivariate analysis	High expression	-
Cervical cancer 22	Up- regulated	-	-	-	-	-
Nephroblastoma 28	Up- regulated	Tumor size, NWTS stage, histopathological type	-	-	-	-
Glioma 29	Up- regulated	TNM stage	OS	K-M plot	High expression	-
Breast cancer 26	Up- regulated	Tumor size, TNM stage, differentiation, lymphatic metastasis	OS	K-M plot	High expression	an AUC of 0.930
Breast cancer 25	Up- regulated	Distant metastasis, lymphatic metastasis, TNM stage	-	-	-	-
Osteosarcoma 30	Up- regulated	-	OS	K-M plot	High expression	-
Pancreatic cancer 18	Up- regulated	-	OS	K-M plot	High expression	-
Endometrial carcinoma 19	Up- regulated	FIGO stage, lymph node metastasis	OS	K-M plot	High expression	an AUC of 0.639

DSS: Disease-Specific Survival; DFS: Disease-Free Survival; OS: Overall Survival; AJCC pathological stage: American Joint Committee on Cancer pathological stage: K-M plot: Kaplan-Meier plot; AUC: Area Under the Curve; TNM stage: Tumor-Nodes-Metastasis stage; FIGO stage: International Federation of Gynecology and Obstetrics stage; NWTS stage: National Wilms Tumor Study stage.

## Abbreviations

LncRNA: Long noncoding RNA
SOX21-AS1: SRY-Box Transcription Factor 21 Antisense Divergent Transcript 1
AD: Alzheimer's Disease
EMT: Epithelial-Mesenchymal Transition
TCGA: The Cancer Genome Atlas
ceRNAs: Competing Endogenous RNAs
HCC: Hepatocellular Carcinoma
PI3K: Phosphoinositide 3-Kinase
AUC: Area Under the Curve
ROC: Receiver Operating Characteristic
GBM: Glioblastoma Multiforme
LGG: Low-Grade Glioma
LC: Lung Cancer
LUSC: Lung Squamous Cell Carcinoma
CESC: Cervical Squamous Cell Carcinoma and Endocervical Adenocarcinoma
LAML: Acute Myeloid Leukemia
THCA: Thyroid Carcinoma
PAAD: Pancreatic Adenocarcinoma
DSS: Disease-Specific Survival
DFS: Disease-Free Survival
OS: Overall Survival
ACC: Adrenocortical Carcinoma
UVM: Uveal Melanoma
SARC: Sarcoma
STAD: Stomach Adenocarcinoma
THYM: Thymoma
STES: Stomach and Esophageal Carcinoma
DFI: Disease-Free Interval
READ: Rectum Adenocarcinoma
PFI: Progression-Free Interval
HNSCCs: Head and Neck Squamous Cell Carcinomas
